# Overview on Techniques to Construct Tissue Arrays with Special Emphasis on Tissue Microarrays

**DOI:** 10.3390/microarrays3020103

**Published:** 2014-04-17

**Authors:** Ulrich Vogel

**Affiliations:** Institute of Pathology, University Hospital, Eberhard-Karls-University, Liebermeisterstrasse 8, 72076 Tuebingen, Germany; E-Mail: ulrich.vogel@med.uni-tuebingen.de; Tel.: +49-7071-29-82265; Fax: +49-7071-29-2258

**Keywords:** tissue microarrays, techniques, paraffin, OCT

## Abstract

With the advent of new histopathological staining techniques (histochemistry, immunohistochemistry, *in situ* hybridization) and the discovery of thousands of new genes, mRNA, and proteins by molecular biology, the need grew for a technique to compare many different cells or tissues on one slide in a cost effective manner and with the possibility to easily track the identity of each specimen: the tissue array (TA). Basically, a TA consists of at least two different specimens per slide. TAs differ in the kind of specimens, the number of specimens installed, the dimension of the specimens, the arrangement of the specimens, the embedding medium, the technique to prepare the specimens to be installed, and the technique to construct the TA itself. A TA can be constructed by arranging the tissue specimens in a mold and subsequently pouring the mold with the embedding medium of choice. In contrast, preformed so-called recipient blocks consisting of the embedding medium of choice have punched, drilled, or poured holes of different diameters and distances in which the cells or tissue biopsies will be deployed manually, semi-automatically, or automatically. The costs of constructing a TA differ from a few to thousands of Euros depending on the technique/equipment used. Remarkably high quality TAs can be also achieved by low cost techniques.

## 1. Introduction

With the advent of new histopathological staining techniques (histochemistry, immunohistochemistry, *in situ* hybridization) and the discovery of thousands of new genes, mRNA, and proteins by molecular biology the need grew for a technique to compare many different cells or tissues on one slide in a cost effective manner by sparing labor and staining consumables and with the advantages of equal staining conditions and the possibility to easily track the identity of each specimen: the tissue array (TA).

Basically, a TA consists of at least two different specimens per slide.

**Figure 1:**
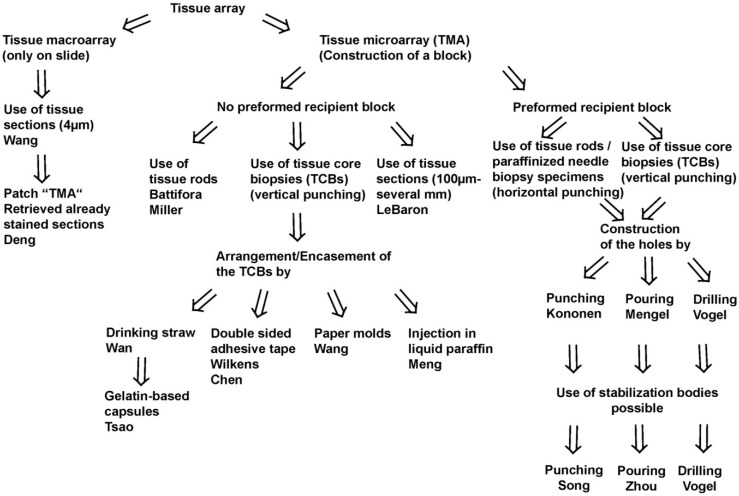
Schematic overview on techniques to construct tissue arrays.

**Figure 2:**
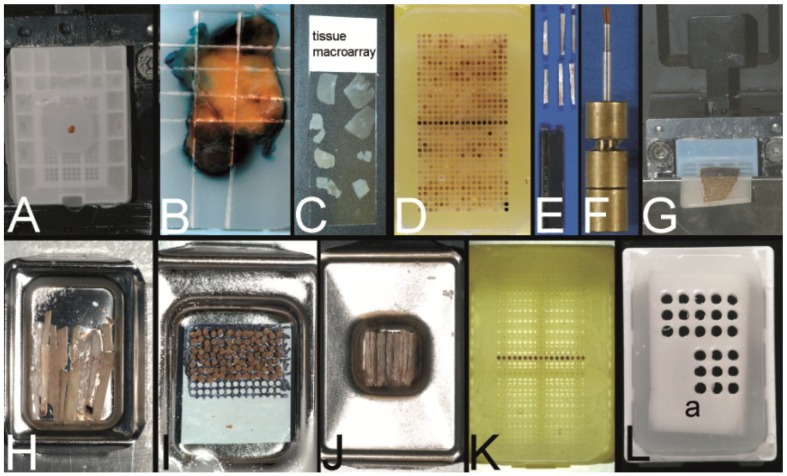
Overview on techniques to construct tissue arrays. (**A**–**C**) Tissue **macro**array: Paraffin block with one small biopsy (**A**). Paraffinblock with tumor of a resection specimen: The paraffin block may be scratched to get several small fragments of the tumor out of one section (**B**). Sections of eight different specimens are arranged on one slide to create a tissue **macro**array (**C**). (**D**) A typical paraffin tissue **micro**array (TMA) with 561 paraffin tissue core biopsies (Computer numerical control (CNC) predrilled recipient block, manually deployed paraffin tissue core biopsies 0.6 mm in diameter). (**E**) Tissue rods trimmed with a razor blade as material to construct TMAs. (**F**) Paraffin tissue punch (1 mm in diameter, Beecher Instruments, Inc., Sun Prairie, WI, USA) with a paraffin tissue core biopsy protruding at the tip. (**G**) Thick paraffin section (100 µm) being cut on a rotary microtome as material to construct TMAs. (**H**) Tissue rods in a routine steel embedding mold to be poured with paraffin to get paraffin tissue layers, which can be stacked to produce a TMA. (**I**) Paraffin tissue core biopsies standing upright on a double-sided adhesive tape, which is mounted on a routine x-ray film placed in a standard steel embedding mold. (**J**) Many different thick paraffin sections are stacked to create a paraffin TMA. (**K**) A CNC predrilled recipient block, which will become a paraffin TMA (completely filled in **D**). (**L**) A predrilled paraffinized agar block (a) embedded in a standard paraffin block to function as stabilization body.

Two fundamental techniques to create a TA may be separated (Figure 1, Figure 2A–C): The tissue **macro**array with different tissue or cellular materials being arranged directly on slides as imprints/suspensions of cells or sections of tissue or cell blocks [1], and the tissue **micro**array (TMA) with the construction of a tissue microarray block from which a lot of sections can be cut (Figure 2D).

With the exception of the recently published patch TMA by Deng *et al*. by which cores of already stained and retrieved tissue sections are arranged on a slide the term TMA comprises the construction of a TMA block in the following [2].

Since the, probably, first description of a TMA for histochemistry by Lilie in 1965, several different techniques to construct a TMA and many synonyms for TMA were published until now [3]. A schematic overview on the techniques may be found in Figure 1. A more comprehensive overview in chronological order is presented in Table 1.

TMAs may differ in:

- the kind of specimens (cells (e.g., from effusions), cell lines, tissues (needle core biopsies, resection specimens)).
- the technique to prepare the specimens to be installed (knife for tissue rods, tissue punches for tissue core biopsies, microtomes for sections (Figure 2E–G).
- the arrangement of the specimens (haphazardly or oriented, distance of the specimens).
- the number of specimens installed (2–10,000).
- the dimension of the specimens (0.1 mm–5 mm).
- the embedding medium depending on the physicochemical property of the specimens (Optimal cutting temperature (OCT) compound for frozen material, paraffin for formalin fixed material, resins for formalin, or glutaraldehyde fixed material).
- the technique to construct the TMA itself (not preformed recipient block, preformed recipient block with or without a stabilization body).
- the aim of the TMA (e.g., Battifora: multipurpose TMA, segmented TMA, theme oriented segmented TMA, clinically defined segmented TMA [4]; for more details see also Kajdacsy-Balla *et al.* [5]).

**Table 1:** Chronological compilation of techniques to construct tissue arrays (TA) and TA applications (only the first name of publications or patents cited).

Year	Author	Name of the technique/tissue microarray
1965	Lilie	Special blocking and trimming procedure for cross sections of multiple small tubular structures [3]
1986	Battifora	Multitumor (sausage) tissue block (MTTB): wrapped fixed tissue rods [4]
1987	Wan	Multi-tissue straw of paraffin embedded tissue cores: drinking straw as encasement [6]
1988	Kraaz	Multiblock control for immunohistochemistry: 4 mm skin biopsy punch modified with a mandrin; cores placed into a warm cast [7]
1988	Rowden	Histocomposites for immunohistological screening of monoclonal antibodies: alignment of the tissue sticks by standard hand-operated cigarette roller; swine casing [8]
1990	Battifora	Checkerboard tissue block: stack of agar plates with embedded fixed tissue rods [9]
1991	Miller	Multitumor (sausage) blocks (MTSBs) as controls for immunohistochemistry: stack of paraffin plates of tissue rods [10]
1994	Press	Multitumor tissue blocks: tissue strips arranged in rows separated by a layer of parafilm [11]
1994	Rose	Multiblock slides for teaching: Kraaz punching method, grid pattern by careful hand positioning [12]
1994	Sundblad	Simplified multi-tissue blocks (SMB): Wedge shaped tissue rods removed from the surface of a paraffin donor block and further processed similar to the Miller technique [10,13]
1997	Petrosyan	Multispecimen tissue blocks: Multichambered (“honeycomb”) plastowax dividers prepared with a rubber mold [14]
1998	Kononen	**Tissue microarray (TMA)**: punching paraffin tissue cores; arrangement in a Cartesian coordinate system; development of a manual tissue arrayer [15,16]
2000	Gillett	Multiple tissue core array: Tissue cores punched with a 11-gauge core cut needle and installed into a recipient block with preformed holes, which were punched with a 13-gauge needle. Even grid by using the back of a standard R. A. Lamb processing cassette as a 34-hole template [17]
2000	Chan	Multitissue spring-roll control block: On-slide multitissue controls for immunohistochemistry [18]
2001	Hoos	Tissue microarrays using cell lines and frozen tissue microarrays (OCT) [19]
2001	Schoenberg	Frozen tissue microarray: OCT embedding medium [20]
2002	Packeisen	Tissue microarray: Kononen technique without using the tape transfer system [21]
2002	Badve	Multi**organ** tissue blocks [22]
2003	Mengel	Tissue microarrays constructed with poured holes and a double melt procedure [23]
2003	Vogel	Tissue microarrays constructed with predrilled ordinary steel embedding molds [24]
2003	Hidalgo	Small format tissue array: manual construction using a bone marrow aspiration needle [25]
2003	Wang	Tissue macroarray by arranging section fragments on different slides [1]
2003	Matysiak	Tissue microarrays automatically constructed with a simple method: semi-automated Kononen tissue arrayer [26]
2003	Wilkens	Tissue microarrays constructed with a double sided adhesive tape [27]
2004	Schnetz	Robotic tissue arrayer using the punching technique of Kononen in combination with positive and negative pressurized air [28]
2004	Vogel	TMA constructed with a microcompound table and a drill grinder [29]
2004	Vogel	Tissue microarrys constructed with a computer numerical control (CNC) drilling machine [30]
2004	Dan	Tissue microarray constructed with a common microscope [31]
2004	Pan	High density tissue array (manual construction): conventional 16-gauge bone marrow biopsy trephine apparatus to puncture the paraffin blocks [32]
2005	Vogel	Tissue microarrays with paraffin tissue core biopsies of 0.43 mm in diameter [33]
2005	Howat	Resin tissue microarrays [34]
2005	LeBaron	Ultrahigh density microarrays of solid samples: stacks of tissue sections [35,36]
2005	Montgomery	Tissue microarrays from suspension cells: paraffin embedding of cell pellets in Eppendorf tubes and punching the cells out of the tube [37]
2005	Datta	Microarrays from needle biopsy specimens: foil templates to reshape paraffinized needle biopsy specimens for further installment into Kononen TMAs [38]
2005	Chen	Tissue microarrays without prefabricating recipient blocks: double sided adhesive tape technique with x-ray film backbone [39]
2005	Meng	Tissue microarrays constructed with the ZM-1 arrayer by injecting the tissue cores in liquid paraffin [40]
2005	Mengel	Tissue microarrays of agar embedded cell lines for on-slide Control [41]
2005	Song	Tissue microarray made of paraffinized agar: stabilization body technique [42]
2006	Vogel	Tissue microarrays using a stabilization body [43]
2006	Vogel	Tissue microarrays filled with a paraffin tissue punch with a countersink [44]
2006	Pires	Tissue microarrays constructed with custom-built needles and double sided adhesive tape technique [45]
2006	Wang	Tissue arrays manually constructed using a hand-made paper mold [46]
2007	Vogel	Tissue microarrays constructed by combining different techniques [47]
2007	Vogel	Tissue microarrays with cracks cured with a soldering iron and adhesive tape [48]
2007	Zhou	Tissue microarray technology for frozen pathological samples: agar as stabilization body [49]
2007	Jiang	Microarray group: Different sections of small TMAs arranged on one slide [50]
2008	Vogel	Tissue microarrays constructed with evenly long core biopsies created with a cutting board and a cutting board arrayer [51]
2008	Szekeres	Tissue micro-array builder for pouring TMAs [52]
2009	Vogel	Cast recipient blocks for paraffin tissue microarrays using conventional steel embedding molds (“top pin tissue arrayers”) [53,54]
2009	McCarthy	Checkers of prostate biopsy specimens installed in wax templates for TMA construction [55]
2010	Vogel	Tissue microarrays constructed from needle biopsy specimens by combining the drilling technique with the adhesive tape technique [56]
2010	Tsao	Gelatin-based capsules for frozen TMA construction [57]
2011	Fridman	Vertical clustering re-arrangement technique for prostate needle biopsies [58]
2011	Shebl	Mechanical pencil tips as paraffin and tissue punches [59]
2011	McCarthy	Improvement of the checker technique by punching the prostate biopsies out of the checkers with the Kononen technique (Beecher tissue arrayer) [60]
2012	Pilla	Implementation of a barcode-driven error control of the design and execution of a TMA by using the laboratory information system [61]
2012	Yang	HT-1 tissue arrayer: punching the holes of the recipient block in one action; negative pressure for removing air bubbles [62]
2012	Choi	Dot grid paper on surface of a recipient block to structure the TMA [63]
2013	Deng	Patch TMA: TMA on a slide using cores of retrieved already stained sections [2]
2013	Shi	Tissue rods: punched parallel to the donor block surface to ensure equal length of the rods and the tissue of interest in every section to be cut [64]
2013	Foda	Modification of Shebl’s technique with mechanical pencil tips as punches [65]
2013	Garcia-García	Inexpensive self-made tissue punches useful in paraffin TMAs [66]
2013	Zanini	Homogeneous distribution of cells or spheres in cell blocks used for TMA construction by shaking [67]

A TMA can be constructed by arranging the tissue specimens in a mold and subsequently pouring the mold with the embedding medium of choice without the need for a prefabricated recipient block (Figure 1, Figure 2H–J) [4,6,9,10,35]. Tissue rods as well as tissue core biopsies (TCBs) or tissue sections may be used (Figure 2E–G) [4,6,10,35]. To prevent the TCBs from tumbling and to structure the TMA, different methods were designed like an encasement in a drinking straw [6], the use of a double sided adhesive tape (Figure 2I) [27,39], paper molds or even the injection of the cores in already liquid paraffin [40,46].

In contrast preformed so-called recipient blocks consisting of the embedding medium of choice have punched, drilled or poured holes of different diameters and distances in which the cell or tissue biopsies will be deployed manually, semi-automatically, or automatically (Figure 1, Figure 2K) [15,23,24,52,53]. Concerning paraffin TMAs (PTMAs) an additional melting process may be performed to get a strong contact between the paraffin of the PTMA and the paraffin tissue core biopsies (PTCBs) installed [4,6,23]. The use of a stabilization body preferably made of agar may facilitate this melting process by allowing a one step complete melting procedure (Figure 2L) [42,43].

The costs of constructing a TMA differ from a few to thousands of Euros depending on the technique/equipment to be used. Remarkably high quality TMAs can be also achieved by low cost techniques [27,29].

In the following, the milestones in the development of the TMAs and some minor modifications of the techniques are described chronologically. Furthermore, the criteria to choose the right technique in a certain setting are discussed and low cost methods presented.

## 2. The Development of the TMAs in the Course of Time

### 2.1. The Multitumor (Sausage) Tissue Block Invented by Battifora (1986) [4]

**Figure 3:**
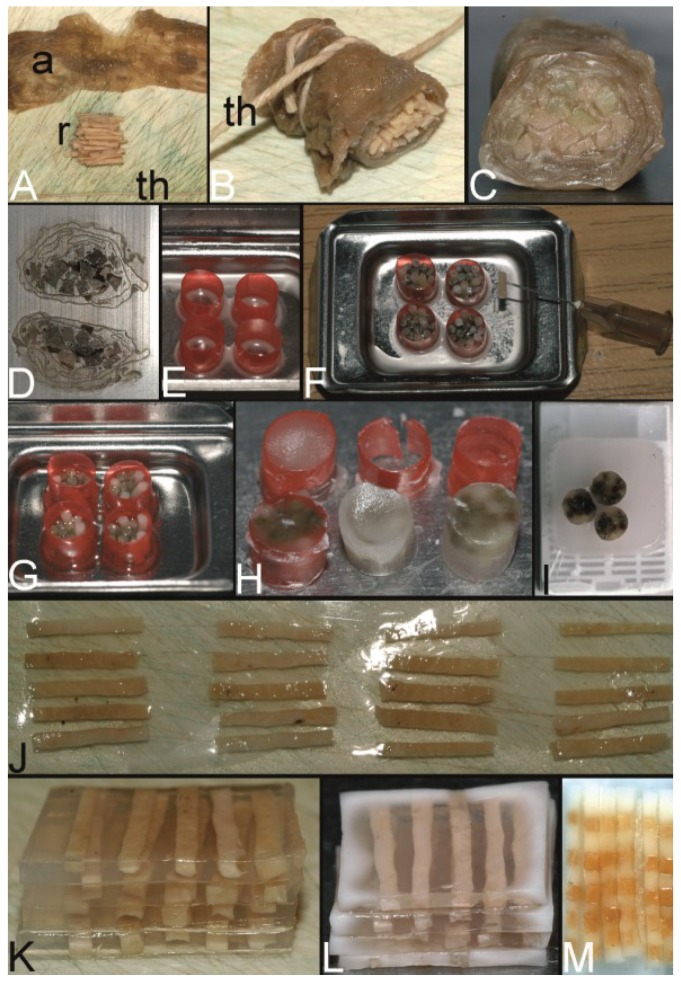
The trail-blazing precursor techniques of the modern tissue microarrays. (**A**–**D**) The multitumor (sausage) tissue block by Battifora. Fixed tissue rods (r) stacked like log piles were tightly wrapped in amnion (a) (instead of Battifora’s small intestine of small mammals) (**A**), secured by thin thread (th) (**B**), paraffinized (**C**), trimmed and cast into a standard paraffin block (**D**). (**E**–**I**) The multi-tissue straws of Wan *et al*. Sections of an ordinary red plastic drinking straw were fixed in a standard steel embedding mold by a little amount of paraffin (**E**) to enhance the installment of paraffin tissue core biopsies (PTCBs) (own modification) (one PTCB on the tip of the needle) (**F**). These filled straws were melted to glue the PTCBs together (**G**). After resolidification the plastic encasement was removed (**H**) and the composed PTCBs embedded in a standard paraffin block (**I**). (**J**–**M**) The checkerboard tissue block by Battifora and Mehta. Stripes of fixed tissue were poured into agar plates (**J**), the plates were stacked (**K**), paraffinized (**L**), trimmed and cast into a standard paraffin block. The cut surface with the brown tissue rods and the white surrounding agar. (**M**) (Note: alignment of the rods not as precise as by Battifora and Mehta).

In 1986, Battifora published his technique to construct a multitumor (sausage) tissue block to test new antibodies on about a hundred tissues on one slide [4]. Tissues of interest were excised by knife from paraffin tissue blocks, deparaffinized in xylene, and rehydrated using a descending alcohol series (100%–50%). These rehydrated tissues were trimmed with a razor blade to rods of about 10 mm in length and of an average cross-sectional area of 1 mm^2^ (Figure 3A). About 100 different rods were tightly wrapped in small intestine of small mammals, such as rabbits (Figure 3B), and routinely reparaffinized (Figure 3C). More than a 1000 5 µm thick sections could be prepared from such multitumor tissue blocks (Figure 3D).

Although being a milestone in the development of TMAs this method appeared to be too laborious especially due to the unnecessary deparaffinization process prior to construct the TMA. Furthermore the TMA must be constructed very carefully to relocate a single probe. The wrapping of the tissue rods is not so easy (own experience). Therefore, Battifora himself improved this technique a few years later.

### 2.2. The Multi-Tissue Straws of Wan et al. (1987) [6]

With their publication in 1987 Wan *et al.* introduced the punching technique for retrieving the paraffin tissue material (the PTCBs) to be later installed in PTMAs [6].

The tip of a 16 gauge syringe needle was removed and the new edge resharpened to get an instrument resembling a miniature cork borer (Figure 8A,B in Section 2.7). Mounted on a plastic syringe tissue cores could be removed from paraffin blocks and extruded from the needle with a wire stylet. These tissue cores were stored in vials (Figure 12I in Section 4) creating a tissue library or directly placed into an ordinary plastic drinking straw (6.3 mm in diameter) as encasement (Figure 3E,F). These straws with an average of 24 tissue cores were then melted to get a firm contact between the paraffin of the tissue cores (Figure 3G). After resolidification the plastic straw casing was removed (Figure 3H). One or more tissue straws could then be embedded in a paraffin block and sectioned (Figure 3I). By using a marker core with a certain pattern of staining or structure, the orientation in every straw was facilitated. With this technique about 120 individual tissue samples could be evaluated on a standard slide.

The published method of punching the paraffin tissue of interest (the PTCBs) was trailblazing and is used in nearly every technique for PTMAs today. By using PTCBs the possibility of establishing tissue banks of a small volume was introduced. Furthermore, this technique avoided the unnecessary deparaffinization process before assembling the specimens as proposed by Battifora, therefore preceding current technology. Wan *et al.* also mentioned the possibility of different core sizes and addressed the problem of sampling errors due to tissue heterogeneity. The compilation of several tissue cores to elongate the core was also described.

Due to the uniformity of the core size and the use of a marker core the accurate tissue identification within the straws was facilitated in contrast to the technique of Battifora.

However, the stabilization of relatively few PTCBs during the melting process by the straw appeared to be too laborious for wide-spread application. Moreover, the installment of the PTCBs into the straws needs good training (own experience) that the PTCBs do not topple down in the encasement.

In 2010, Tsao *et al.* adopted the Wan technique to construct low cost frozen tissue microarrays by using sectionable gelatin-based capsules for arranging the punched frozen tissue [57].

### 2.3. The Checkerboard Tissue Blocks by Battifora and Mehta (1990) [9]

Although sticking to the formerly described complex deparaffinization technique this publication of Battifora and Mehta in 1990 became a footstep by introducing the alignment of the tissue specimens in a Cartesian coordinate system (checkerboard pattern) [9].

By using a multi-blade knife of disposable microtome knives and different-sized spreaders tissue rods of uniform thickness and square cross sections were cut out of fresh or dewaxed tissue. These rods were placed into the rectangular grooves of an aluminum tissue embedding mold and covered with fluid agar 3% at 60 °C (Figure 3J; manual alignment without an aluminum mold). The solidified agar plates were stacked (Figure 3K) and placed in a perforated metal cassette for paraffin embedding. 

By aligning the specimens in a Cartesian coordinate system the exact relocation of a single probe was easy to manage and led the way to current techniques. Battifora and Mehta already anticipated the possibility of mass screening of tissue samples for new prognostic markers probably by the use of automatic robotic screening and the use of the TMA for interlaboratory quality assessment.

They also mentioned the compilation of several short rods within the grooves to elongate the rods.

However, due to the complex construction process with deparaffinization and agar embedding this technique did not get wide-spread acceptance.

**Figure 4:**
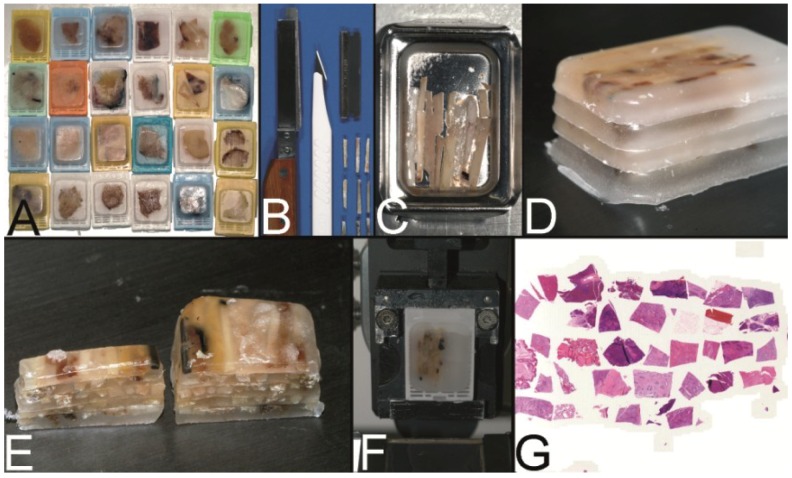
The Miller technique—tissue rods. (**A**) Multiple paraffin blocks used as donor blocks. (**B**) For cutting out the paraffinized tissue rods a trimming knife, a scalpel or a razor blade may be used. (**C**) Alignment of the tissue rods in one layer in a routine steel embedding mold to be cast into a paraffin tissue plate. (**D**) Stacking of different paraffin tissue plates. (**E**) Trimming of the stacked plates. (**F**) Sectioning of the embedded stacked plates e.g., for use as positive controls in immunhistochemistry. (**G**) Stained section of the tissue microarray (Hematoxylin Eosin).

In 1991, Miller advanced the technique of Battifora and Mehta by omitting the dewaxing step and the agar embedding [10]. Miller cut already paraffinized tissue into rods (Figure 4B), assembled the rods in one layer in an ordinary steel mold to melt these rods to plates (Figure 4C) and stacked these plates after resolidification of the paraffin (Figure 4D) to get a PTMA. This low cost, simple and robust technique is till now in use especially to create PTMAs for positive controls in immunohistochemistry.

### 2.4. The Tissue Microarray by Kononen et al. (1998) [15]

The breakthrough of the TMAs leading to world wide use especially in the setting of translational research and medicine was achieved by a technique coined as “tissue microarray” which was developed in a cooperation project by the groups of Guido Sauter at the Institute of Pathology of the University of Basel (Switzerland) and of Olli Kallioniemi at the National Human Genome Research Institute in Bethesda (Maryland, USA). This technique was published in 1998 in Nature Medicine by Juha Kononen as first author and may be called the Kononen technique in the following [15]. This publication may represent the numerous well-known publications of these groups.

By constructing the “tissue microarray” the authors combined the punching technique of Wan *et al.* with the precise alignment and easy relocation of the tissue specimens (the PTCBs) of the “checkerboard tissue block” of Battifora and Mehta [6,9].

**Figure 5:**
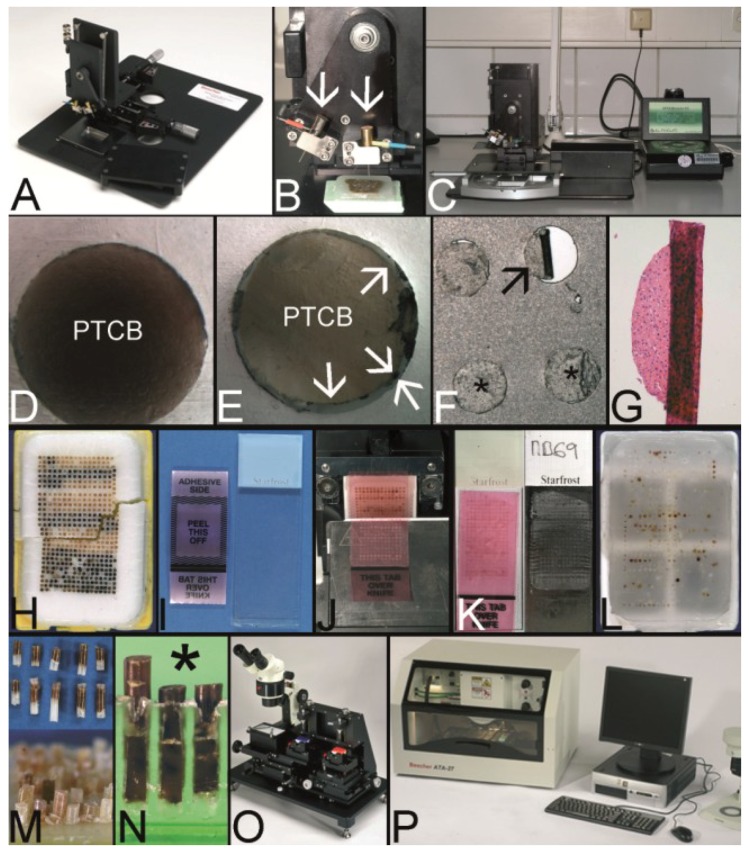
The Kononen technique coined as tissue microarray—punching the holes of the preformed recipient block and the tissue of interest. (**A**) The manual tissue arrayer (MTA-1) developed by Kononen *et al*. and produced by Beecher Instruments Inc., Sun Prairie, WI, USA. (**B**) The turret of the manual tissue arrayer allowing the switch between the two paraffin punches (arrows) (the punch for the paraffin tissue core biopsies (PTCBs) has a somewhat larger inner diameter). (**C**) Tissue arrayer with a motorized stage (Alphelys, Plaisir, France). (**D**) PTCB within the hole of the recipient block: at least one half of the PTCB with a good contact to the surrounding paraffin of the recipient block. (**E**) PTCB within the hole of the recipient block: missing contact to the surrounding paraffin of the recipient block (arrows). (**F**) Paraffin section with some PTCBs with a good contact to the surrounding paraffin (asterisk). One PTCB with a missing contact is rolled (arrow). (**G**) Rolled PTCB after staining with limited evaluation. (**H**) Cracked paraffin tissue microarray at cooling before sectioning. (**I**) Paraffin tape transfer system (Instrumedics Inc., St. Louis, MO, USA) consisting of a tape and a special slide coated with a resin. (**J**) The tape is mounted on the surface of a paraffin tissue microarray block to be loaded with the section. (**K**) The mounted section is transferred to the slide. After UV polymerization of the resin the section sticks to the slide; the tape can be removed after incubation in a solvent. (**L**) Deeply cut paraffin tissue microarray with thinning out of the unevenly long PTCBs. (**M**) Routinely punched PTCBs demonstrating the different length, which is due to the different thickness of the donor tissue. (**N**) Cross section of a paraffin tissue microarray (PTMA): Holes of the recipient block filled with more than one PTCB to avoid thinning in deeper sections. The PTCBs protrude the surface of the PTMA. Surface of the paraffin block (asterisk) (**O**) Tissue arrayer with a reflecting microscope to facilitate the detection of the best punching location on the donor block (Veridiam Oceanside, CA, USA). (**P**) Fully automated tissue arrayer (Beecher Instruments Inc., Sun Prairie, WI, USA).

Kononen *et al.* developed a machine in cooperation with S.B. Leighton (inventor according to US patent), the manual tissue arrayer (Beecher Instruments Inc, Sun Prairie, WI, USA) (Figure 5A), which consisted of two slightly different sized tissue punches (Figure 5B, arrows) mounted on a vertically movable “precision guide” which itself was fixed vertically to a horizontal xy stage. With the recipient punch, which is slightly smaller in diameter than the donor punch, the holes in the recipient block, the later PTMA, were constructed. The PTCBs were punched out of so-called donor blocks, *i.e.*, routinely fabricated paraffin tissue blocks, by using the donor punch and transferred into the holes of the recipient block. Punches of different diameter (e.g., 0.6 mm, 0.8 mm, 1.0 mm, 2.0 mm) were available. As many as 1000 PTCBs could be installed into a 45 × 20 mm recipient block in a perfect Cartesian coordinate system which made the relocation of the PTCBs very easy.

A disadvantage of this method—if the word disadvantage may be even used in the light of the great success of this technique—is the missing melting procedure of the PTMA after the deployment of the PTCBs in contrast to the methods of Battifora, Mehta and Wan (Figure 5D,E) [6,9]. A strong contact between the paraffin of the PTMA and the PTCBs is decisive at cutting and floating the PTMA section on the waterbath. If the section is more than 3 µm thick (own unpublished data) there may be some rolling and folding of the PTCBs in the waterbath (Figure 5F), which miss good contact to the surrounding paraffin. In consequence, such PTCBs have a small contact area to the slide (Figure 5G), cause an increased mechanical resistance during the washing and staining procedures, may float off the slide and will be lost for evaluation therefore reducing the efficacy of the PTMA technique.

Kononen *et al.* also mentioned melting of the PTMA surface to ensure easier sectioning, but warned of moving of PTCBs in the PTMA during melting.

Kononen *et al.* tried to achieve the strong contact of the paraffin of the PTMA and the PTCBs by pressing PTCBs somewhat larger in diameter into smaller holes. Such a procedure may avoid the folding of the PCTBs. However, firstly, such a good fit cannot be achieved for all PTCBs (Figure 5E) and can only be ensured by using not deformed tissue punches.

Secondly, especially high density PTMAs with a hundreds of PTCBs may crack during the cooling procedure before sectioning because of the high tension in the block (Figure 5H) [68]. This tension may be caused by the increased volume of the PTCBs in comparison to the smaller holes. Of course, cracking of a paraffin block is not restricted to Kononen-PTMAs.

To avoid this cracking and some other general problems of sectioning like the disruption of the section on the waterbath, Kononen *et al.* advised the use of the paraffin tape transfer system (Instrumedics Inc., St. Louis, MO, USA) (Figure 5I–K). By mounting a tape on the surface of a PTMA fixed in a microtome clamp a section can be cut at room temperature and will adhere without any folding or cracking on the tape (Figure 5J). This section may be transferred to a slide coated with some resin. After polymerization of the resin by UV light the section will strongly adhere on the slide and the tape can be removed by a solvent (Figure 5K). The disadvantage of this tape system may be the price for the UV lamp and the consumables (about 2,500 €) and the resin itself, which may lead to some problems at fluorescence *in situ* hybridization [68]. Furthermore, according to Hoos and Cordon-Cardo the lowest degree of tissue damage was seen without using the adhesive transfer tape [19]. Catchpoole *et al.* describe a higher incidence of nonspecific staining in immunohistochemistry by using the tape method [69]. For more details on loss of cores see also the review of Pinder *et al.* [70].

The problems of unevenly long PTCBs (Figure 5L–N) or the submerging of PTCBs in the holes of the PTMA which might reduce the efficacy of the TMA technique may be solved by installing as many PTCBs as needed to fill the holes completely [19]. However, the PTCB may be pulled out by the microtome knife or may be bent during the cutting procedure, if the PTCB overtops the surface of the PTMA too much (own experience).

The installment of cores into 2–3 holes per case will minimize a probable sampling problem and the problem of tissue loss due to unevenly long PTCBs and due to rolling and folding of PTCBs [19].

The original Beecher manual tissue microarrayer was improved in a little while to become a computer numerical control (CNC) arrayer with still manual (Figure 5C) or fully automatic transfer of the PTCBs (Figure 5P, automated tissue arrayer ATA-27, Beecher Instruments, Inc. [71,72]) [26].

Furthermore an arrayer was designed with a reflecting microscope to improve the selection of the tissue from the donor block (e.g., VTA-100 Tissue Arrayer (about 55,000 US$), Veridiam, Oceanside, CA, USA [73]) (Figure 5O).

Based on the punching technique of Kononen, Schnetz *et al.* invented a robotic tissue arrayer using negative and positive pressurized air to guide and improve the automatic punching process (Oridis Biomed Forschungs- und EntwicklungsGmbH, Graz, Austria) [28].

The Galileo CK family of semiautomatic tissue arrayers also applies the Kononen technique (Integrated Systems Engineering S.R.L., Milano, Italy [74]).

In 2012 Yang *et al.* presented their HT-1 tissue arrayer by which the holes of the paraffin recipient block are punched out in one action comparable to the technique of Song (see below) [42,62]. This may be one of the fastest methods to create a preformed recipient block. Furthermore, Yang designed a very inventive method to extrude the air between the PTCBs and the holes.

### 2.5. The PTMA by Mengel et al. (2003) [23]

In 2003 Mengel *et al.* reintroduced the melting as a two step procedure into the construction process of PTMAs as originally described by Battifora, Mehta, and Wang to achieve a strong contact between the PTCBs and the surrounding paraffin and to prevent tensions in the paraffin block [6,9,23]. 

Moreover, Mengel *et al.* transferred the cost-effective pouring of the holes of the recipient block, which was already described for frozen TMABs by Hoos and Cordon-Cardo, to paraffin TMAs (PTMAs) [19,23]. This patented procedure is currently licensed by Zymed (San Francisco, CA, USA) or Zytomed (Berlin, Germany) to produce, e.g., customized PTMAs (MaxArray System) as a commercial service [23,75].

**Figure 6:**
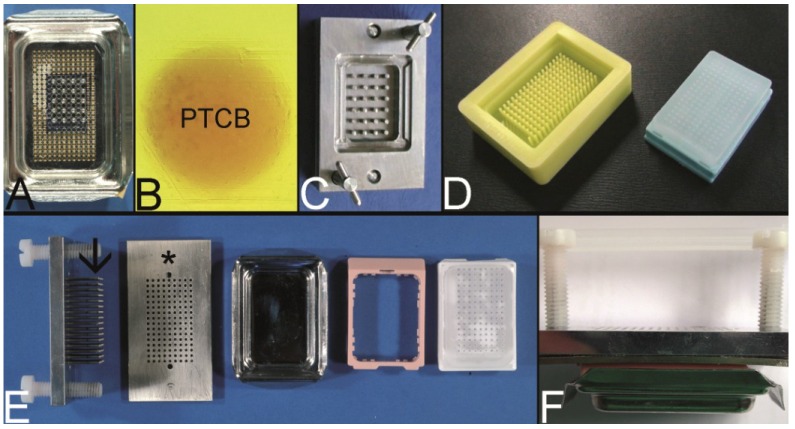
The Mengel technique—pouring the holes of the preformed recipient block. (**A**) Routinely used steel embedding mold with many holes in the bottom through which steel pins are pushed to work as spacers for the holes of the recipient block (for demonstration only 20 steel pins inserted). (**B**) Perfectly melted paraffin tissue core biopsy (PTCB) in a paraffin tissue microarray (PTMA). (**C**) Tissue arrayer for pouring the holes of the recipient block as patented by Szekeres *et al.* (**D**) Tissue arrayer made of rubber for pouring the holes of the recipient block (Unitma, Seoul, South Korea). (**E**,**F**) Top pin tissue microarrayer as designed by Vogel. Steel pins (arrow) fixed to a metal plate are pushed through a perforated plate (asterisk) and inserted from above into a routinely used steel embedding mold (composite in **F**).

In brief, as disclosed in the patent, 60–120 cylinder pins with a diameter of e.g., 1.5 mm were driven into an aluminum block in a Cartesian coordinate system. These pins fit into the holes of the bottom of a modified conventional embedding mold (Figure 6A). After solidification of the paraffin, which was poured into the embedding mold, the cylinder pins were withdrawn resulting in a PTMA blank with up to 120 holes.

This blank was inserted in a second conventional embedding mold and the holes were filled with PTCBs. By applying heat from the bottom of the mold the filled PTMA was melted up to 80% of the height of the block. After resolidification overhead heat was applied for melting the 20% rest of the paraffin to ensure a complete melting of the PTMA.

The equipment for this two-step melting procedure cannot be bought commercially. Customers of Zymed receive a tissue punch to retrieve the tissue specimens of interest and send the PTCBs to Zymed to construct the PTMA.

A great advantage of the Mengel technique is the strong contact between the PTCBs and the surrounding paraffin (Figure 6B) with nearly no loss of PTCBs (<1%) due to folding and rolling rendering the paraffin tape transfer system unnecessary [23]. Unaddressed by Mengel *et al.* the problem of unevenly long PTCBs. Probably only one PTCB may be installed in one hole due to the melting process. By routinely using PTCBs of different length some PTCBs may be lost in deeper sections of the PTMA. Moreover, the Mengel system is designed for 96 PTCBs per array in commercial service, which is less than the number of PTCBs which could be already achieved by Wan *et al*. (120 PTCBs) [6].

A steel mold to cast the holes of the recipient block which is slightly different to that of Mengel was patented by Szekeres *et al.* in 2008, and can now be purchased at Thermo Scientific as Thermo Scientific™ Lab Vision™ Tissue Microarray (TMA) Builder (Waltham, MA, USA [76]) or at 3DHISTECH (Budapest, Hungary [77]) as manual TMA kit (Figure 6C) [52].

Furthermore, a modification of the mold made of rubber is distributed by Unitma (Seoul, Korea [78]) under the brand of Quick-Ray Mold Kit (170 holes, 1.0 mm in diameter, 500 US$/each) (Figure 6D).

Another modification of this technique was introduced by Vogel as top pin tissue arrayer which can be used with ordinary steel embedding molds to cast the holes of the recipient block (Figure 6E,F) [54].

### 2.6. The PTMA by Wilkens and Chen et al. (2003, 2005) [27,39]

Probably, the simplest technique to construct a PTMA was patented in 2003 by Wilkens and published as an apparently second independent invention in 2005 by Chen *et al.* [27,39].

**Figure 7:**
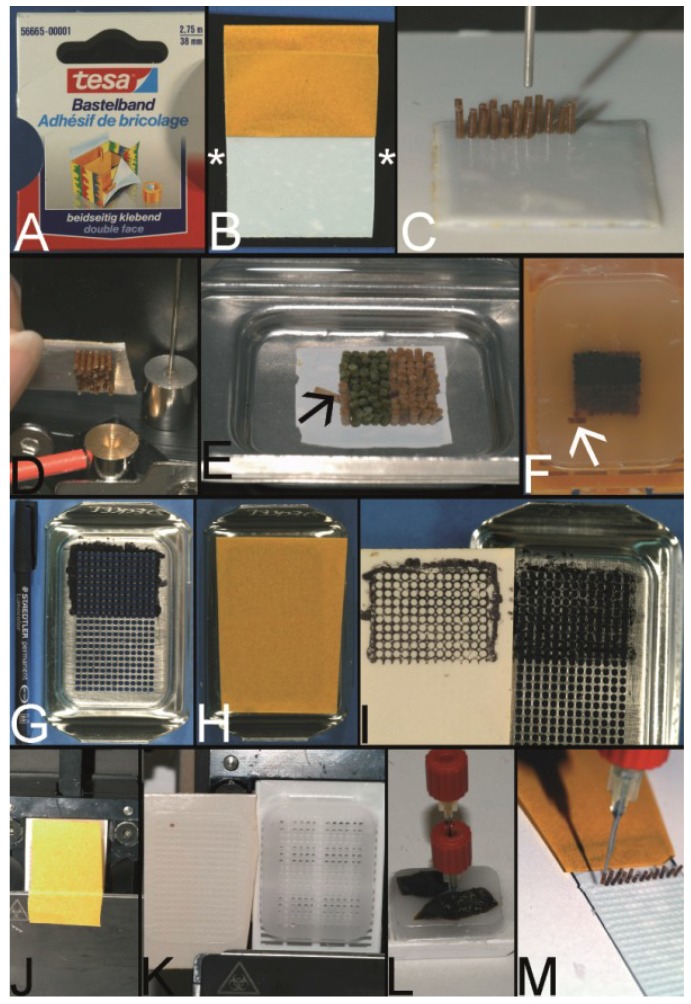
Wilkens and Chen technique—double sided adhesive tape to fix the PTCBs without the need for a preformed recipient block. (**A**) Example for a double-sided adhesive tape to fix the paraffin tissue core biopsies (PTCBs). (**B**) White double-sided adhesive tape with a brown protection paper mounted on a standard black x-ray film (asterisk). (**C**) PTCBs arranged in a Cartesian coordinate system on the double-sided adhesive tape. (**D**) Strong contact between the PTCBs and the double-sided adhesive tape—no PTCB falls off. (**E**) Melting of the PCTB-adhesive tape-x-ray-film-sandwich in a standard steel embedding mold. Do not heat over 65 °C, otherwise the adhesive tape may shrink and destroy the PTMA. Note that one PTCB toppled down (arrow). Not paraffin, but the paraffinized tissue must be in contact with the adhesive tape. (**F**) PTMA after resolidification and removal of the adhesive tape-x-ray-sandwich. Note the tumbled PTCB (arrow). (**G**) Routinely used steel embedding mold with many holes (see also Figure 6A) in the bottom is painted with a standard permanent marker. (**H**) The double-sided adhesive tape is fixed to the painted bottom of the mold. (**I**) After removing the tape from the bottom of the mold the grid of the mold is transferred to the tape and can be used to structure the PTMA. (**J**) The double-sided adhesive tape is fixed to a recipient block with preformed holes, which is mounted on a microtome clamp. (**K**) After cutting a 5–10 µm thick section of the preformed recipient block is fixed to the adhesive tape. (**L**,**M**) PTCBs can now be transferred manually from a paraffin donor block to the grid of the double-sided adhesive tape.

According to the technique of Wan *et al.* PTCBs were punched out of ordinary paraffin tissue blocks and transferred to a double sided adhesive tape (Figure 7A) mounted, e.g., on a piece of regular x-ray film (Figure 7B). The PTCBs were manually aligned in a Cartesian coordination system like in the Battifora/Kononen technique (Figure 7C). The glue of the adhesive tape held the PTCBs in place (Figure 7D) and in an upright position especially when the PTCB-tape-x-ray film sandwich was put in an ordinary steel embedding mold and filled with fluid paraffin to construct the PTMA (Figure 7E).

A disadvantage of this technique may be the use of only one PTCB per spot whereby the PTMA may loose some cores in deeper sections as already discussed. Furthermore, the manual alignment of the cores may be not so precise as with the Beecher tissue arrayer making the evaluation of the stained sections more complicated. When using PTCBs smaller than 0.6 mm in diameter the fluid paraffin has to be poured into the mold very carefully in order not to incline the PTCBs which may also make the evaluation of the stained section difficult (own unpublished experience).

Nonetheless, Wilkens and Chen proved that the construction of a PTMA with a Cartesian alignment of the PTCBs is possible without a prefabricated recipient block in a very cost-effective way.

A modification of this technique was published by Wang *et al.* in 2006, using a hand-made paper mold instead of an adhesive tape to keep the PTCBs in line [46].

Furthermore, different systems to define a grid on the tape were designed. Pires *et al.* used a translucent adhesive tape and put a piece of paper with a printed grid under the tape [45]. Vogel developed two different systems to get some kind of a grid on the tape: One system with a marker-painted metal grid to transfer the ink to the tape (Figure 7G–I); the other system to glue sections of predrilled recipient blocks on the tape (Figure 7J–M).

The technique of Wilkens and Chen is also well suited for curing cracked PTMAs by arranging the broken parts onto the adhesive tape and consecutive melting.

### 2.7. The Predrilled PTMA by Vogel (2004) [29,79]

**Figure 8:**
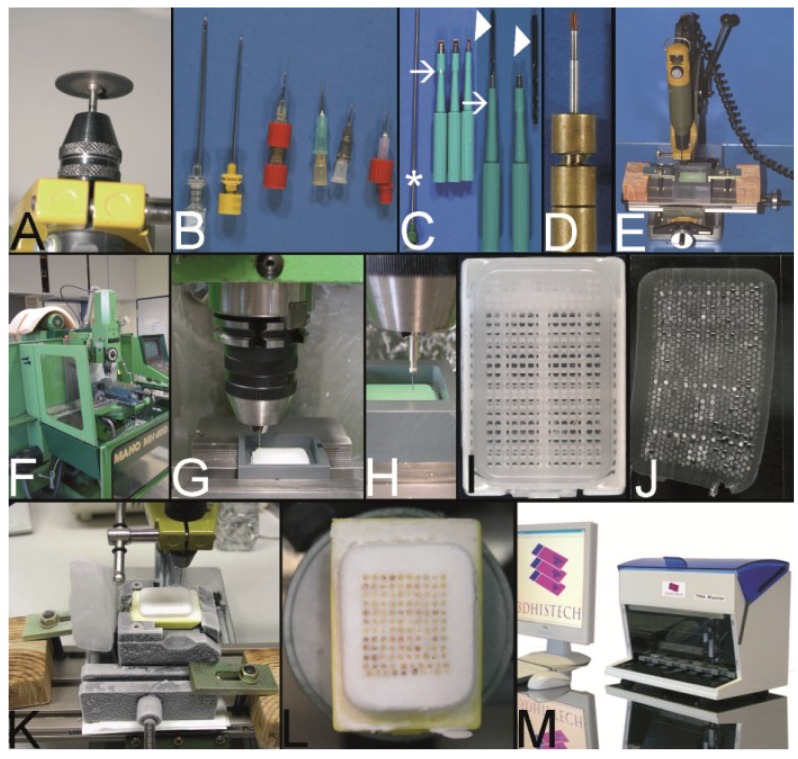
The Vogel technique—drilling of the holes of the recipient block. (**A**) Drill grinder in a drill stand with a cutting disk (Proxxon GmbH, Föhren, Germany). (**B**) Tissue punches of different inner diameters (0.3 mm to 1.0 mm) constructed out of routine needles. In case needles are commercially not provided with a stylet, wires or other needles may be used as stylets. Infusion caps (red pieces) (**C**) Skin punches of different inner diameters (1–5 mm; Kai Industries). The stylet of a bone marrow biopsy needle (asterisk) can help to push the PTCBs out of the skin punches after the narrow (arrows) was widened manually with a drill bit (arrowhead). Nowadays, the skin punches are also provided with a built-in stylet; however, this stylet may be easily jammed by paraffin and may break (own unpublished observation). (**D**) Resharpened commercial paraffin tissue punch (Beecher Instruments, Inc.), which was waste material of a TMA core facility after breakage of the tip of the cannula. (**E**) A water bath made of polyvinylchloride mounted on a microcompound table (x-y table) which is fixed to a drill stand equipped with a drill grinder (Proxxon GmbH, Föhren, Germany). A standard paraffin block is fixed within the water bath for drilling of the holes. (**F**–**H**) Computer numerical control (CNC) drilling machine. The water bath is fixed to a bench vice of the CNC drilling machine. The holes of the recipient block are drilled under water (cooling effect and floating off the paraffin debris). (**I**) Paraffin recipient block perfectly drilled by the CNC drilling machine. (**J**) Section of a paraffin recipient block with a honeycomb pattern to enlarge the number of installed PTCBs. Perfect drilling by the CNC machine. (**K**) Drilling the holes of a recipient block made of optimal cutting temperature (OCT) medium for frozen TMAs. The OCT block is mounted on a microtome clamp, which is fixed to the microcompound table on the drill stand. The clamp was cooled in a freezer before drilling. (**L**) Filled frozen TMA mounted on the clamp of a freezing microtome. (**M**) Fully automated tissue arrayer using the drilling technique (TMA Grand Master, 3DHistech, Budapest, Hungary).

In 2004 Vogel presented a method to construct PTMAs by using a conventional drill grinder, a microcompound table and a drill stand which could be purchased in every hardware store for less than 300 € (Proxxon GmbH, Föhren, Germany) [29,79]. In brief, the tips of routinely used hypodermic needles were cut with a cutting disk using the drill grinder and resharpened as proposed by Wan *et al*. (Figure 8A,B) [6]. Skin biopsy punches (Kai Industries, Seki, Japan) (Figure 8C) or commercially available paraffin tissue punches (Figure 8D) were also used to retrieve the PTCBs. The holes of the prefabricated recipient block were drilled in a Cartesian alignment into a standard paraffin block (Figure 8E). The recipient block was fixed in a water bath of polyvinylchloride (PVC) (Figure 8E). The PTCBs were punched from donor blocks and manually transferred to the holes of the recipient block with the optional use of an illuminated magnifying glass, which may be found in every laboratory of pathology.

With this low cost equipment high densitiy PTMAs could be constructed with more than 600 precisely arranged PTCBs per standard paraffin block. Like with the Kononen technique a melting step was primarily not included causing some rolling and folding of PTCBs at sectioning when the paraffin tape transfer system of Instrumedics Inc. was not used.

Vogel modified this technique by using a computer numerical control (CNC) drilling machine (Figure 8F–I) for creating up to 2500 holes 0.3 mm in diameter into a standard paraffin block to achieve the highest number of PTCBs per PTMA to this day when using a prefabricated recipient block technique [80,81]. With the CNC drilling machine also special arrangements of the holes (e.g., in a honeycomb pattern, Figure 8J) were easily possible to enhance the number of PTCBs to be installed [82]. The CNC-drilled holes displayed the highest quality in comparison to all other drilling and punching techniques.

In the meantime the drilling technique to create the holes in the recipient block is incorporated in an automated PTMA construction machine (e.g., TMA Grand Master) by 3DHISTECH (Budapest, Hungary) (Figure 8M).

The drilling technique is also applicable for the construction of frozen TMAs (Figure 8K,L).

A tissue arrayer applying the drilling technique is also manufactured by Mr. Mirlacher, University of Hamburg. Apparently, there is only one hint for this arrayer being published in a subordinate clause [83]; the arrayer is only constructed on demand.

### 2.8. The PTMA of Paraffinized Agar by Song (2005) [42]

Independently invented by Mengel *et al*., Vogel, Yan *et al*., and Song, Song was apparently the first inventor and consistently got the patent on a method to use stabilization bodies (e.g., of paraffinized agar) as recipient blocks [42,84,85,86].

**Figure 9:**
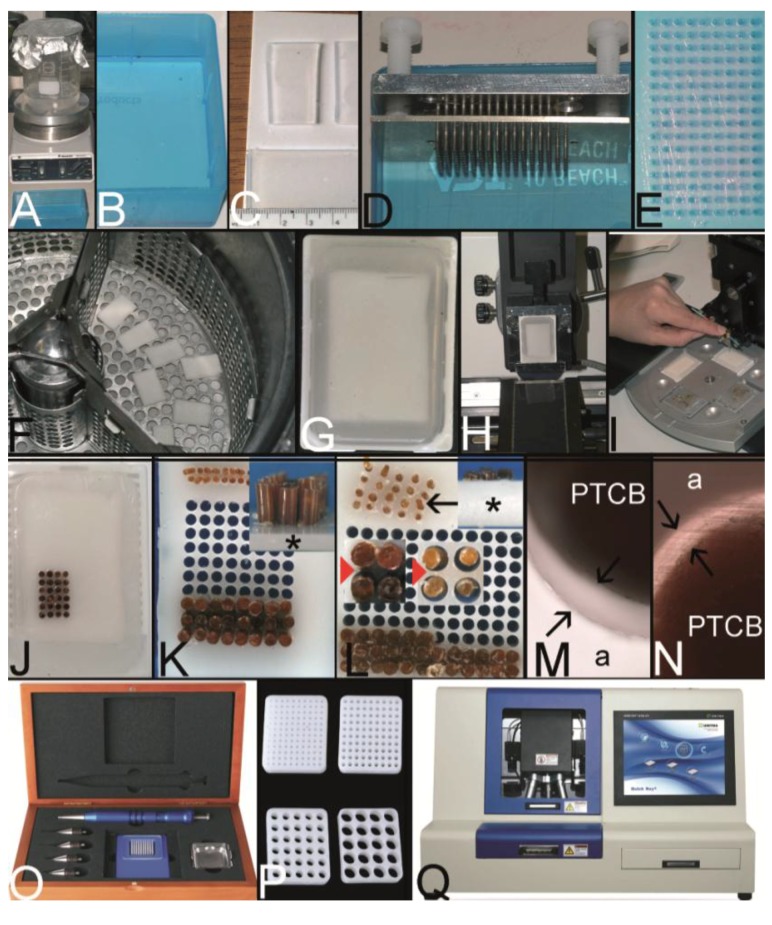
The Song technique—agar stabilization bodies for a one step fully melting procedure. (**A**) Boiling agar 2% like in the molecular biologic laboratory for gels or in the kitchen. (**B**) The liquid agar is poured into the lid of a pipette box (waste material). (**C**) The solidified agar, which can be simply released from the mold is cut into plates of desired dimension by a scalpel. (**D**) Pouring the holes into an agar plate: This top pin tissue arrayer is placed into liquid agar. (**E**) After solidification of the agar the pins are withdrawn from the agar by turning the screws (**D**). (**F**) The agar plates (with or without preformed holes) are paraffinized in a standard automatic tissue processor. (**G**) The agar plate (agar stabilization body) is poured into a paraffin block. (**H**) Before drilling the holes into such a paraffin block and/or before filling the holes of a cast agar plate (**D**,**E**) sectioning of the block is recommended until the agar plate is in contact with the block surface. (**I**) The holes of the stabilization body can also be punched, e.g., with a manual tissue arrayer. (**J**) Agar stabilization body cast into a paraffin block with the holes being filled with PTCBs. (**K**) Agar plates can also be used and filled with PTCBs as a stand alone and may be cast into a paraffin block after melting. Note, this agar plate (asterisk) is thin (see insert) and gives stabilization only for one PTCB per hole. The advantage of this thin plate is the better release of air bubbles at melting. (**L**) A thick agar plate nearly completely surrounds the PTCBs at the entire length (see also insert (upper right corner) with a thick stabilization body (asterisk)). The holes can be filled with more than one PTCB to ensure an equal length and to prevent the thinning of the PTCBs in deeper sections. PTCBs of different diameters can be installed into the holes (inserts with arrowheads). The holes of the agar plate can be also constructed by punching manually with some more or less precise arrangement of the cores (arrow). Different diameters of the holes are possible. (**M**) Gap (arrows) between a PTCB and the surrounding agar stabilization body (a) before melting. (**N**) Gap (arrows) filled with paraffin after melting (a agar stabilization body). (**O**) Quick Ray manual tissue microarrayer set (Unitma, Seoul, South Korea). (**P**) Agar stabilization bodies of 1 mm, 1.5 mm, 3 mm, and 5 mm (Unitma, Seoul, South Korea) (**Q**) Fully automated tissue arrayer of Unitma (Seoul, South Korea) constructing PTMAs with preformed stabilization bodies.

In brief, Song poured a block of agar, paraffinized it and punched out the holes of the later PTMA by using a Cartesian aligned grid of punches. These holes could be filled with PTCBs of an adequate diameter. Then the filled agar recipient block was put into an ordinary mold and filled with liquid paraffin to create the PTMA whereby the agar block stabilized the PTCBs and prevented them from tumbling. After resolidification, the PTCBs were in perfect contact with the surrounding paraffin (Figure 9N).

This system can be purchased from Sakura (Tokyo, Japan [87]; various websites for Japan, Europe and America) which sells the system under the brand of Tissue-Tek Quick-Ray, or from Unitma (Figure 9O, Quick Ray manual tissue microarrayer (full set: 3,500 US$/set, Seoul, South Korea [78]). A disadvantage of the Quick-Ray system may be the limitation to about 170 PTCBs per PTMA. But nonetheless, this is a powerful and simple technique to construct PTMAs especially within the aspect of an incorporated one step full melting procedure.

In the meantime, an automated tissue arrayer which punches out the PTCBs of the recipient block and transfers them to the holes of the preformed recipient block is now sold by Unitma (Seoul, South Korea) for about 78,000–98,000 US$ depending on the version (Figure 9Q).

Besides, this technique is well suited for a low cost construction of PTMAs because the agar stabilization bodies can be easily fabricated in every laboratory of pathology (Figure 9A–H) [88]. The holes of these agar blocks can be created by drilling, pouring (Figure 9D,E) or even punching with the Beecher tissue arrayer (Figure 9I). Especially with the drilling technique as many holes as needed and every diameter of the holes necessary may be provided if a low cost technique is favored.

### 2.9. Ultrahigh Density Microarrays of Solid Samples by LeBaron et al. (2005) [35,36]

In 2005, LeBaron published a technique, also called the cutting edge matrix assembly, to construct PTMAs with the highest number of specimens up to day, *i.e*., up to 10,000 different specimens per block [35,36].

**Figure 10:**
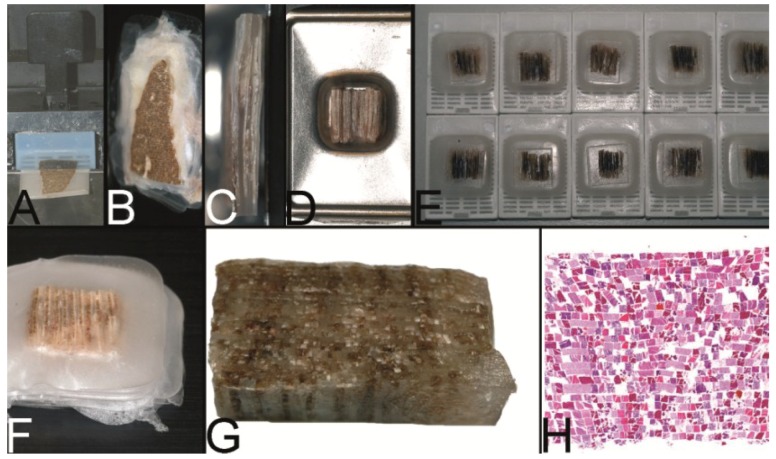
Le Baron technique—PTMA made of thick sections. (**A**) Routine paraffin tissue block used as a donor block fixed to the clamp of a rotary microtome to cut sections 100 µm thick. (**B**) 100 µm thick sections of different donor blocks being stacked (primary stack) and glued together by gently warming (30–40 °C). (**C**) Cut surface of the trimmed primary stack of thick sections. (**D**) Cross sections of different primary stacks being arranged in a routine steel embedding mold and cast into a paraffin block. (**E**) Several paraffin blocks with cross sections of a lot of different primary stacks. (**F**) Secondary stack of 100 µm thick sections of the blocks displayed in E. (**G**) Trimmed secondary stack before being cast into a paraffin block. (**H**) Hematoxylin-Eosin stained section of the trimmed secondary stack. The correct arrangement of the sections of the primary and secondary stack does not seem to be so easy to perform to get a Cartesian grid. Furthermore, entrapped air bubbles might cause difficulties.

To achieve such a high number of specimens LeBaron *et al*. avoided the punching technique, which can be applied only to a diameter of the PTCBs equal or greater than 0.3 mm due to the stability of the tissue punches (own unpublished experience). The tissue specimens were cut as plates by knife or microtome with a thickness of about 100 µm (Figure 10A). These tissue plates were melted (Figure 10B) or glued (superglue, *i.e.*, methacrylat) together to receive a primary stack and sectioned again, until 3D plates as secondary stacks (Figure 10F) resulted. Of course, this technique was only useful for tissues with the cells of interest being homogeneously distributed. One spot of tissue in a section reached only 100 µm^2^.

Although this technique is brilliant there may be sometimes some difficulties in gaining such 100 µm thick plates of tissue, e.g., by cracking (unpublished own experience). Furthermore the exact arrangement of the plates and the melting of the plates may not always be easy (Figure 10G,H; own unpublished experience).

### 2.10. Combined Techniques for PTMA Construction

#### 2.10.1. Punching Technique/Drilling Technique/Pouring Technique Combined with the Double Sided Adhesive Tape Technique of Wilkens/Chen (Vogel, 2007) [47,89]

**Figure 11:**
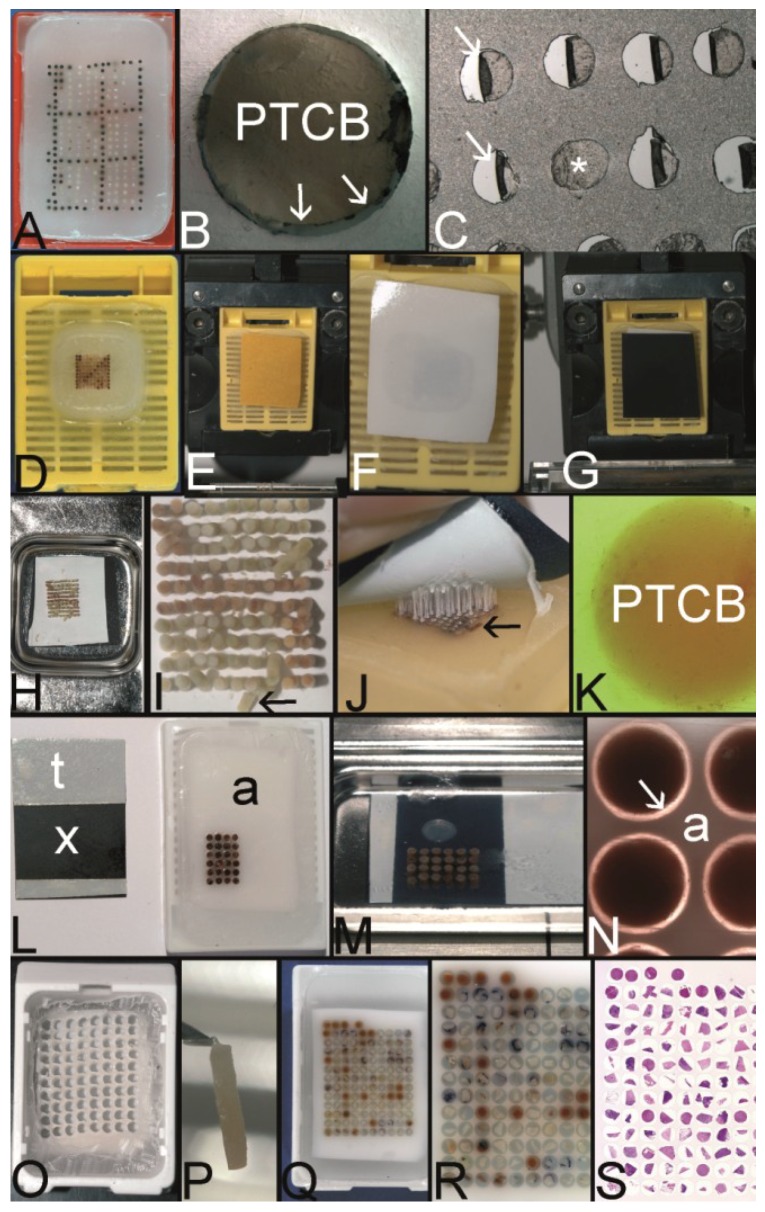
Combined techniques. (**A**) PTMA of an external TMA laboratory constructed with the Kononen technique. (**B**) Large gap between the PTCB and the surrounding paraffin of the recipient block. (**C**) Section of this PTMA with perfect (asterisk) and rolled PTCBs (arrows) if the paraffin tape transfer system of Instrumedics, Inc., is not used. (**D**) Predrilled paraffin recipient block filled with 100 PTCBs 0.43 mm in diameter. (**E**) The PTMA is fixed to the clamp of a rotary microtome and cut to get a smooth surface. A double sided adhesive tape with a brown protective sheet is attached to the surface of the PTMA to get into contact with the PTCBs. (**F**) The PTMA with the white double sided adhesive tape after removal of the protection sheet. (**G**) An x-ray film is attached to the double-sided adhesive tape to stabilize the tape at melting. (**H**) The PTMA-adhesive tape-x-ray film-sandwich is melted in a standard steel embedding mold (note: Do not heat over 65 °C, in order not to shrink the adhesive tape.). (**I**) Melted PTMA with the PTCBs standing upright and in position due to the adhesive tape. Of course, if a hole is filled with more than one PTCB, the PTCB without contact to the tape will topple down (arrow). (**J**) After resolidification the double-sided adhesive tape-x-ray-film-sandwich is removed from the surface of the PTMA demonstrating the strong adhesion of the PTCBs to the glue of the adhesive tape. Note the toppled down PTCB (arrow) (**K**) After resolidification the PTCB displays a strong contact to the surrounding paraffin. (**L**) Black x-ray film (x) with two strips of the white double sided adhesive tape (t) and an agar stabilization body (a) cast into a standard paraffin block and filled with some PTCBs. (**M**) After fixing the x-ray film-tape-sandwich to the surface of the agar stabilization body (paraffinized agar binds to the tape) this sandwich is melted in a standard steel embedding mold. The PTCBs are held in position by the stabilization body and not by the adhesive tape. This very small gap between the x-ray film and the stabilization body facilitates the flow of the liquid paraffin into the gaps between the PTCBs and the agar stabilization body. This small gap is ensured by the tape and the x-ray film. Without the x-ray film-tape-sandwich the short and/or small PTCBs may fall out of the holes of the stabilization body at melting. (**N**) After resolidification the gap (arrow) between the PTCBs and the agar stabilization body (a) is perfectly filled with paraffin; this secures a very low number of rolled PTCBs at sectioning. (**O**) Predrilled agar stabilization body cast into a paraffin block with a bottomless plastic cassette (Tissue-Tek Paraform Sectionable Cassette System, Sakura, Tokyo, Japan) (Look from above). The surface of the agar stabilization body is fixed to a x-ray film-double sided adhesive tape-sandwich. (**P**) A paraffinized breast needle biopsy specimen (PNBS), which was punched out of the donor block, melted to remove the adhering paraffin surplus and resolidifed at the tip of a small needle. This PNBS can now be installed into the hole of a stabilization body. (**Q**) Agar stabilization body filled with PNBSs after melting, resolidification and removal of the x-ray film-tape-sandwich. (**R**) Higher magnification of the surface of the PTMA (**Q**) demonstrates a perfect contact between the PNBSs and the agar stabilization body. (**S**) A Hematoxlin-Eosin stained section of the PTMA filled with PNBSs.

The Kononen/Beecher technique using the manual or automated tissue arrayer may be the mostly used technology for constructing PTMAs word-wide. However, the already mentioned rolling and folding of PTCBs or the cracking of the PTMA at sectioning may be a problem if the paraffin tape transfer system is not used (Figure 11A–C). Of course, these problems also occur when the holes of the recipient block are drilled or poured. The rolling and folding could be prevented by combining the aforementioned techniques with a melting step, especially with the technique of Wilkens/Chen (Figure 11D–K) [27,39,47,89].

Generally three techniques may exist for melting a PTMA: a partial melting procedure (e.g., 18 min, 58 °C in an oven), a one step complete melting procedure and a two step melting procedure as introduced by Mengel [23].

The partial melting technique is a rapid and simple procedure, which may be used very often word-wide. However, extreme care has to be taken for PTMAs with PTCBs of less than 1 mm in diameter that the PTCBs do not tumble during the melting. The larger the diameter of the PTCBs the less the probability of tumbling. The PTCBs have to be stabilized by the still solid paraffin in the upper parts of the PTMA.

In contrast to own experience the two-step melting procedure is said to be easy to perform, e.g., by using an *in situ* hybridization platform and a heating lamp. Firstly, the bottom half of the block is melted by the platform, secondly after cooling the upper half is melted by a heating lamp (see above).

Probably the easiest way to get a fully melted PTMA is to cut a filled PTMA (Figure 11D) on a microtome to get a smooth surface, to fix the double sided adhesive tape-x-ray film-sandwich of Wilkens/Chen to the smooth surface of the PTMA (Figure 11E–G) and to melt this sandwich (Figure 11H,I). Independently of the diameter the PTCBs are fixed in place and in an upright position during the melting by the tape. There is no time limit which has to be strictly obeyed at melting like in the partial or two-step melting procedures. The only disadvantage of this kind of melting is that PTCBs may tumble down if more than one PTCB is installed in one hole of the PTMA or if the tissue of the PTCB has no contact to the tape.

This disadvantage may be cured by using an agar stabilization body as recipient block as described by Song. (Figure 11L–N) [42]. 

#### 2.10.2. Stabilization Body Technique Combined with the Wilkens/Chen Double Sided Adhesive Tape Technique (Vogel) [27,39]

Such a combination of the techniques is especially useful when constructing PTMAs by using paraffinized needle biopsy specimens (PNBSs) (Figure 11O–S). Such PNBSs can be put into punched or drilled holes of an agar stabilization body, which is mounted on a double-sided adhesive tape. After filling the holes, the stabilization body (recipient block) can be melted in a one step procedure without tumbling of the upright PNBSs (Figure 11Q–S). The double-sided adhesive tape-x-ray film-sandwich creates a reversible bottom to the stabilization body and, therefore, prevents the PNBSs to fall out of the holes during filling or melting of the PTMA. This technique may be easier and faster than the checker technique as described by McCarthy *et al.* [55,60].

#### 2.10.3. Rod Technique of Miller Combined with the Punched Preformed Recipient Block of Kononen (Shi *et al.*, 2013) [64]

The paraffin tissue core biopsies (PTCBs) as described by Wan and Kononen are punched vertical to the surface of the donor paraffin block [6,15]. The disadvantages of these PTCBs are the uneven length as discussed above and the unknown tissue composition in the depth. These obstacles can be overcome by punching or dissecting parallel and not perpendicular to the surface of the donor block. These tissue rods, as they were called by Shi *et al.*, apparently in remembrance of the Miller technique have a definite equal length and the tissue of interest at least near the former surface in all sections to be cut [64]. These tissue rods were prepared using a unique sampling tool for which a Chinese patent exists. The rods were installed in a prewarmed softened paraffin recipient block with holes being drilled with a steel needle and arranged in a Cartesian coordinate system.

#### 2.10.4. The Microarray Group—A Combination of the Tissue Macroarray and the Tissue Microarray (Jiang *et al.* 2007) [50]

In 2007, Jiang *et al.* presented a technique, which they called the microarray group [50]. They arranged sections of different small PTMAs on one slide thereby greatly enhancing the TMA effectiveness. Up to 2534 PTCBs 0.6 mm in diameter could be examined on one standard glass slide. This technique was also presented by Vogel arguing that smaller PTMAs may also increase the flexibility of the TMA technique. Probably, it would be more reasonable to construct smaller PTMAs, e.g., with subsets of tumors instead of creating one large tumor PTMA [90].

## 3. How to Select the Appropriate Technique for Constructing TAs?

The selection of the appropriate technique for constructing TAs depends on several factors, which may also interfere with each other (Table 2).

**Table 2:** Factors influencing the choice of the tissue array (TA) technique.

Factors influencing the choice of the tissue array (TA) technique
Intent of the TAs (e.g., as positive control for routine immunohistochemistry, for translational research)
Physical property of the tissues/embedding medium (frozen material for frozen TMAs, formalin fixed paraffin embedded tissue for PTMAs, paraformaldehyde fixed material for resin TMAs)
Number of TCBs to be installed in the TMAs
Dimension of tissues to be evaluated (e.g., needle biopsy specimens, resection specimens, cell blocks)
Frequency of the construction of TAs
Money to be spent

The most important question which has to be answered at first, belongs to the intended use of the TA. It is the purpose, which determines the choice of the material (fresh frozen, formalin fixed paraffin embedded, paraformaldehyde), the number of the TCBs to be installed in a TMA, and the need for a TMA. A compilation of the techniques may be found in Table 3.

The simplest form of a TA is the tissue **macro**array, *i.e*., the array only on the slide. For most applications, however, the construction of a TMA is needed.

If a TMA should serve as a positive control in immunohistochemistry with less than about 30 specimens per TMA, the construction of PTMAs is advised according to several well functioning zero or low cost techniques (e.g., Miller (tissue rods), Wilkens/Chen (tissue cores)), which don’t need a prefabricated recipient block [10,27,39].

**Table 3:** Selection of the appropriate paraffin TA technique.

Specifications	Appropriate Techniques
Few slides necessary Few specimens (<10)	Tissue **macro**array (the array on the slide) [1]
Many slides necessary Few specimens (20–30) No precise arrangement necessary No preformed recipient block	Miller: tissue **micro**array (TMA): tissue rods [10] Wilkens/Chen technique: TMA: double sided adhesive tape [27,39] Melting step inherent to the technique
Many slides necessary Few specimens (30–100) Precise arrangement necessary Preformed recipient block more comfortable Manual transfer of tissue cores	Poured, punched or drilled paraffin or paraffinized agar TMAs: commercially available tissue arrayers or ready to use recipient blocks (Mengel, Song, Szekeres, Vogel) [23,42,52,79] Additional melting step with the adhesive tape-x-ray film-sandwich advised [89]
Many slides necessary Many specimens (>100) Precise arrangement necessary Preformed recipient block advised Construction of **only a few** TMAs	Commercial services to construct the TMA Cooperation with a TMA facility at university
Many slides necessary Many specimens (>100) Precise arrangement necessary Preformed recipient block advised Semiautomatic/automatic transfer of tissue cores Construction of **many** TMAs	Commercially available tissue arrayers: e.g., Beecher Instruments Inc., 3DHistech, Veridiam, Unitma, (see above) Additional melting step with the adhesive tape-x-ray film-sandwich advised if not inherent to the technique itself [89]

Although the Wilkens/Chen technique can be used to construct PTMAs with more than 300 PTCBs, it seems more comfortable especially in the setting of translational research to use some preformed poured, punched or drilled recipient blocks when more than 30–50 PTCBs should be installed. These recipient blocks may be pure paraffin blocks or paraffinized agar blocks (stabilization bodies), which are commercially available or can be fabricated by oneself, e.g., with a mold and spacers made of steel or rubber (e.g., Mengel, Song, Szekeres, Vogel) [23,42,52,79]. By applying these systems the PTCBs have to be transferred manually.

For more than 100 PTCBs per PTMA the decision should be made whether the PTCBs should be still transferred manually, semi-manually/semi-automatically with the help of a machine or even automatically. This choice of the appropriate device is of course also dependent on the numbers of PTMAs, which should be constructed in the course of time. If only a few PTMAs are intended for construction, it may be inefficient to invest in an expensive automatic tissue arrayer. Commercial services for construction of PTMAs may also be a choice in this scenario. Furthermore, assistance may be provided by colleagues, e.g., of tissue microarray units of university institutes in terms of collaboration.

Only if multiple PTMAs with a high number of PTCBs are to be constructed the acquisition of a semi-automatic or automatic tissue arrayer may be favorable and cost-effective. Several suppliers may fulfill the needs for such a device (e.g., Beecher Instruments Inc., 3DHistech, Veridiam, Unitma, see above). Probably the most used machine may be the manual tissue arrayer MTA-1 of Beecher Instruments Inc. for about 10,000 Euros.

Of course, possible devices should fit for the embedding medium of choice. Whereas the expensive commercially available tissue arrayers are mostly restricted for paraffin, low cost devices may be also used for frozen TMAs.

Furthermore, the equipment of choice should also provide the appropriate diameter of the PTCBs. Depending on the tissue and the scientific question to be answered the diameter of the PTCBs needed may vary from 0.3 mm (e.g., for homogeneous, densely packed tumors, such as endocrine tumors) to 5 mm (e.g., for skin or mucosal preparations where the epithelial-mesenchymal interface is of interest).

## 4. Low Cost Techniques to Construct TAs of High Quality

In this section some low cost techniques may be recommended for those who do not have access to a tissue microarray facility, e.g., at universities, who want to obviate the waiting time at tissue microarray facilities and who do not want to spend money for commercial services, but who want, nonetheless, TAs of high quality.

As already discussed at point 3, the technique used may be chosen according to the intent of the TA.

If a tissue **macro**array, *i.e*., the array on a slide, is not applicable, a TMA must be constructed.

For frozen TMAs the use of a frozen **agar** stabilization body is strongly recommended with holes being poured or due to low costs preferably drilled [49,79]. Even when the frozen TMA may warm up unexpectedly, the TMA keeps the structure due to the agar.

The technique of Tsao *et al.* using gelatin-based capsules may also be an alternative for low cost frozen TMA construction [57].

**Table 4:** Low cost techniques for the construction of tissue arrays.

Specifications	Appropriate techniques
Few slides necessary Few specimens (<10)	Tissue **macro**array (the array on the slide) [1]
Many slides necessary Few specimens (20–30) No precise arrangement necessary	Miller: tissue **micro**array (TMA): tissue rods [10]
Many slides necessary Few specimens (30–50) Precise arrangement necessary	Wilkens/Chen technique: TMA: double sided adhesive tape [27,39]
Many slides necessary Many specimens (50–300) Precise arrangement necessary	Vogel manual drilling: TMA: Preformed recipient blocks [79] Stabilization body advised [88] Melting with the adhesive tape-x-ray film-sandwich [89]
Many slides necessary Multiple specimens (>300) Precise arrangement necessary	Vogel manual drilling or CNC drilling: TMA: Preformed recipient blocks [79,81] Stabilization body advised [88] Melting with the adhesive tape-x-ray film-sandwich [89]

A prerequisite for a high quality paraffin TMA (PTMA) is a complete melting step in the construction process. Only a complete melting step ensures a firm contact between the PTCBs and the surrounding paraffin of the PTMA which is necessary to avoid rolling and folding of the PTCBs at sectioning as described above (Section 2.5). By this step the paraffin tape transfer system mostly becomes needless. Possible low cost techniques are summarized in Table 4.

A melting step is always included in the techniques working with tissue rods (e.g., Miller) or with PTCBs without the use of a prefabricated recipient block (e.g., Wilkens/Chen: double sided adhesive tape) [10,27,39]. If PTMAs made of tissue rods or PTMAs with a small number of PTCBs may fulfill the task, these techniques are advised with costs being negligible.

If PTMAs with more than 50 PTCBs are needed, a prefabricated recipient block in form of a stabilization body preferably made of agar is favorable to perform a one step complete melting procedure. The holes of these stabilization bodies can be poured before or preferably drilled or punched after the paraffinization process. The most appropriate method to create the holes may be the drilling technique because of the enormous flexibility and the exact arrangement of the holes. By drilling, different diameters of the holes due to the huge amount of commercially available drill bits, different distances of the holes and multiple arrangements of the holes are feasible. Care should be taken to drill through the whole thickness of the stabilization body that the air bubbles can leave the holes during the melting process. Furthermore, the hardware for drilling with costs of less than 300 € is much cheaper than any commercially available pouring system or prefabricated stabilization body. The disadvantage of the pouring systems or the prefabricated stabilization bodies may be the often low number of holes defined by the number of pins of the arrayer.

If more than 300 holes/PTMA are needed, the use of a CNC drilling machine is advised although manual drilling may also achieve this task. CNC drilling machines can often be found in the department of fine mechanics of a larger hospital or any neighboring metal-working company.

Stabilization bodies may also provide the possibility to use more than one short PTCB in one hole in combination with the melting process. The construction of PTMAs with paraffin needle biopsy specimens is also facilitated by using stabilization bodies because the biopsies are held in an upright position during the melting process.

If a stabilization body is not intended to be used the melting can also be done by fixing the PTCBs of a predrilled PTMA to a double sided adhesive tape as described above. However, in such case, the holes of the PTMA can be filled with only one PTCB.

**Figure 12:**
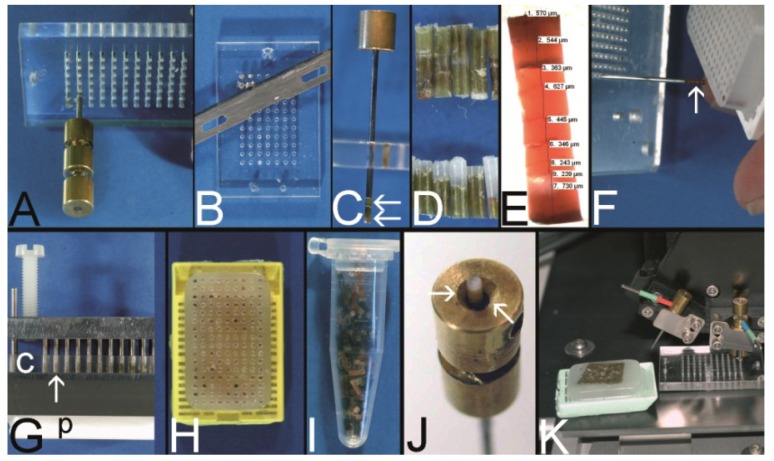
Vogel technique to construct evenly long PTCBs. (**A**) A so-called cutting board is a 4 mm thick plate made of e.g., polymethylmethacrylate (plexiglas^®^) with numerous holes to accommodate PTCBs. The PTCBs can be installed into the plate with a paraffin tissue punch. (**B**) The holes of the cutting board can be filled with as many PTCBs as necessary to achieve a length of 4 mm. The part of the PTCB which surmounts the surface of the cutting board can be easily cut with a used microtome knife and inserted into another hole to spare the sometimes precious tissue. (**C**) The composite PTCB (arrows) can be retrieved from the cutting board with the stylet of the paraffin tissue punch. (**D**) The PTCBs punched out of the donor paraffin blocks are of different length (bottom). After the installment into the cutting board the composite PTCBs are of equals length (top). (**E**) A 4 mm long composite PTCB composed of several very short PTCBs. (**F**) The composite PTCBs (arrow) can be transferred into the holes of the recipient blocks separately. (**G**) A tissue arrayer can also be used to transfer all of the composite cores (arrow) of the cutting board (c) into the holes of the recipient block simultaneously (p plastic cassette of the recipient block. (**H**) A PTMA filled with composite PTCBs by the tissue arrayer. (**I**) PTCB library in an Eppendorf vial as already proposed by Wan *et al*. (**J**) A commercially available paraffin tissue punch (Beecher Instruments, Inc.) was modified with a countersink to facilitate the installment of stored PTCBs into a punch. (**K**) The cutting board can also be used in combination with a tissue arrayer (e.g., the Beecher tissue arrayer).

To overcome the problem of unevenly long PTCBs the use of a cutting board is advised (Figure 12A–F) [91]. Such a cutting board consists of a plate of polymethylmethacrylate (plexiglas^®^) for example with a height of 4–5 mm and with at least one hole with a diameter of the correspondent PTCBs (Figure 12A,B). The plate is reversibly fixed or pressed to a firm base. The hole of the plate is filled with as much PTCBs as needed. The protruding part of the last PTCBs can be cut and inserted into another hole of the plate or stored in a vial for further use to spare the often precious tissue. With a stylet the PTCB composite is released (Figure 12C) and injected into the hole of the PTMA (Figure 12F). The cutting board can also be used to store PTCBs for further deployment into PTMAs in the future with the advantage of an already homogeneous length of the PTCBs.

The installment of the PTCBs into the cutting board can be also accomplished with a Beecher tissue arrayer (Figure 12K).

A very good idea to overcome this problem of unevenly long PTCBs, seems to be the rod technique as proposed by Shi [64], which is similar to the wedge shaped tissue rod technique as described by Sundblad [13]. If the tissue is punched parallel to the donor block surface the length of the rods and the tissue composition is definitely determined. Of interest may be the sampling tool to perform this horizontal tissue acquisition.

In contrast to tissue rods, which can be trimmed with an ordinary knife, PTCBs need tissue punches. The most cost-effective method is to produce tissue punches out of routinely used hypodermic needles by oneself. As described by Wan *et al*. the tip of the needle is cut with a cutting disk and the new end resharpened with the use of a grinding device [6]. As stylet a smaller hypodermic needle may be used. Due to the widespread use of needles in medicine a large variety of different diameters exist. Care has to be taken to remove the grinding dust before using the needle as a punch. In addition to the low costs these punches are normally very stable because of the relative high wall thickness in comparison to commercially available punches. A special tissue punch with a lateral opening 1 mm away from the tip was manufactured out of conventional hypodermic needles (16 and 18 gauge) by Pires *et al*. [45].

Commercially available skin biopsy punches with or without a plunger system may also be used as tissue punches (e.g., kai biopsy punches) (Figure 8C). If a skin biopsy punch without a plunger system is applied, a stylet (e.g., of a bone marrow biopsy needle) has to be used to release the PTCBs from the punch. To get the stylet through the punch the transition of the metal punch and the plastic handle has to be widened by an appropriate drill bit. This drilling can be done manually. A minor disadvantage of the skin biopsy punches is the vulnerability of the very sharp tip.

The tissue punches may also be purchased from the manufacturers of the tissue arrayers (e.g., Beecher Instruments Inc.) and then used manually. Damaged tissue punches may be obtained from tissue microarray facilities for free and can be resharpened for further manual use.

Other construction methods for tissue punches were proposed by Shebl *et al.*, Foda, and Garcia-Garcia *et al*. (e.g., mechanical pencil tips) [59,65,66].

When working with PTCB libraries, *i.e.*, the storage of PTCBs in vials (Figure 12I), a modification of the paraffin tissue punches with a countersink is helpful (Figure 12J) [92]. This countersink can be fabricated with the same drill grinder, which is used for drilling, and a larger cone-shaped precision cutter (e.g., 3–4 mm in diameter; costs: less than 5 €).

## 5. Conclusion

Since 1965, several efficient techniques to construct TAs have been developed to meet the needs of the user. Especially the intended use of the TAs (e.g., positive controls for immunohistochemistry or translational research) may decide what kind of technique (e.g., tissue rods or PTCBs) or whether a low cost technique or a high priced automated system is chosen. Even with low cost techniques high density and precise TMAs can be constructed. It is a pleasure to see what different kind of techniques colleagues all over the word invented to fulfill only one task: To get tissue core biopsies into holes/blocks.
